# Methodological issues on the use of administrative data in healthcare research: the case of heart failure hospitalizations in Lombardy region, 2000 to 2012

**DOI:** 10.1186/s12913-016-1489-0

**Published:** 2016-07-08

**Authors:** Cristina Mazzali, Anna Maria Paganoni, Francesca Ieva, Cristina Masella, Mauro Maistrello, Ornella Agostoni, Simonetta Scalvini, Maria Frigerio, On behalf of the HF Data Project

**Affiliations:** Department of Management Economics and Industrial Engineering, Politecnico di Milano, Milan, Italy; MOX–Department of Mathematics, Politecnico di Milano, Via Bonardi 9, 20133 Milan, Italy; Department of Mathematics, Università degli Studi di Milano, Milan, Italy; Ospedale Uboldo, AO Melegnano, Milan, Italy; AO San Carlo di Milano, Milan, Italy; IRCCS Fondazione S. Maugeri di Lumezzane, Brescia, Italy; De Gasperis Cardiocenter, Niguarda-Ca’Granda hospital, Milan, Italy

**Keywords:** Heart failure, Administrative databases, High dimensional data methods, Comorbidity, Epidemiological studies, Healthcare services utilization

## Abstract

**Background:**

Administrative data are increasingly used in healthcare research. However, in order to avoid biases, their use requires careful study planning. This paper describes the methodological principles and criteria used in a study on epidemiology, outcomes and process of care of patients hospitalized for heart failure (HF) in the largest Italian Region, from 2000 to 2012.

**Methods:**

Data were extracted from the administrative data warehouse of the healthcare system of Lombardy, Italy. Hospital discharge forms with HF-related diagnosis codes were the basis for identifying HF hospitalizations as clinical events, or episodes. In patients experiencing at least one HF event, hospitalizations for any cause, outpatient services utilization, and drug prescriptions were also analyzed.

**Results:**

Seven hundred one thousand, seven hundred one heart failure events involving 371,766 patients were recorded from 2000 to 2012. Once all the healthcare services provided to these patients after the first HF event had been joined together, the study database totalled about 91 million records. Principles, criteria and tips utilized in order to minimize errors and characterize some relevant subgroups are described.

**Conclusions:**

The methodology of this study could represent the basis for future research and could be applied in similar studies concerning epidemiology, trend analysis, and healthcare resources utilization.

## Background

Heart failure (HF) is one of the main causes of morbidity, hospitalization and death in the western world; the costs associated with HF management are relevant and may be expected to increase in the future [1]. Several authors addressed the study of heart failure using administrative data in terms of epidemiology [2–4], outcomes [5–7], contents and quality of the process of care, and costs of treatment [8, 9] and sometimes they linked different type of administrative data [8, 9].

Administrative data represent an unique source of information, whose advantages and disadvantages for the scope of healthcare research had been extensively discussed [10–14]. Briefly, the strong points of these databases are high numbers, universal coverage, and systematic collection of data over time; it means that these databases are probably the best available source for wide epidemiological studies regarding prevalence and incidence of major diagnosis or diseases, and monitoring of trends in utilisation of specific services and procedures. Main limitations are possible lack of accuracy, different coding criteria across individuals and institutions, changing criteria over time, changing in coding system over time, difficulties in linkage and merging of different databases. These limitations are less relevant when the object of the study is a clearly identified event (e.g., acute myocardial infarction, stroke) or procedure (e.g., percutaneous coronary intervention, tracheostomy…) associated with hospitalization, while the picture may be blurred when dealing with conditions characterised by transition from chronic to acute phases and back, as is typically the case of heart failure. For these reasons, working with administrative databases to carry out clinical research requires caution. Many issues, including data quality, criteria for selecting the patients and or the events of interest for the study, methods for recognizing and classifying co-morbidities, and choice of the observation period may affect the results and their interpretation, and must be carefully considered [15, 16]. However, there is a growing interest in using administrative data to address epidemiological and healthcare management research questions, as testified by the high number of papers recently published both in statistical and epidemiological or clinical journals. Administrative data algorithms, based on discharge and procedure codes, are increasingly used for the evaluation of performance, resource needs, and quality of care. A recently published systematic review [17] explicitly distinguishes between administrative data algorithms developed for in-hospital surveillance and those for (external) quality assessment. Beyond epidemiology and process of care, administrative databases could ideally become the most relevant source for broad, all-comers observational studies, that could be put aside evidences built on randomised clinical trials, to get better knowledge about several points: first, are patients enrolled in RCTs representative of general, real world population? Second, are RCTs-based recommendations being applied to different, broader populations? And if yes, are outcomes similar or different with respect to those observed in RCTs? Third, do observational studies on administrative database identify relevant unmet needs that are not addressed by current research? These are maybe too ambitious scopes, but the consideration for “big data” as the new basis for outcome research is growing [18]. In 2011, the Italian Ministry of Health funded a research project submitted by the offices of the Lombardy healthcare system, aimed to build a large and reliable database on patients hospitalized for HF, which should link data on hospitalizations, outpatient service utilization, and drug prescriptions and could be used for epidemiological purposes, cost analysis, risk prediction, and quality of care evaluation, including adherence to recommended treatments. Some papers [19–21] dealing with administrative data focus on the definition of the project dataset design and the treatment of data.

Lombardy is the biggest Italian region, situated in northern Italy, and counting about 10 million residents. There is an universalistic healthcare system, in which the regional offices serve as regulators and payers. Reimbursement to healthcare providers is based in the DRG-IDC9-CM (Diagnosis Related Groups—International Classification of Diseases, 9th Revision, Clinical Modification) version for hospitalizations, and on a specific list of services and drugs for oupatient care. As a consequence, all the healthcare providers and pharmacies send information on services and drugs delivered using standard forms or record layouts to Lombardia Informatica, the company that manages the data warehouse of Lombardy Region, that can attribute all the records to individual subjects utilizing unique identifiers.

Let us point out that in recent years the availability of information technology determined the wide spring out of large administrative electronic databases, in order to estimate incidence, prevalence, and mortality measures and to determine other epidemiological indicators for health problems. Even if the linkage of records from multiple databases could yield reliable information, in Italy the structures of these databases (e.g., databases on hospital discharge forms, vital statistics, outpatient services, Emergency Room [ER] services, drugs prescriptions) are very different, also due to their different purposes, showing a high heterogeneity. Also from an informatics perspective the study of these high dimensional databases needs defining procedures for data management and analysis. It is common to deal with a certain degree of errors that may depend on several reasons such as: issues during the storing procedures, linkage key mismatch and human errors. A huge effort has been performed in the last years to minimise these sources of error, though some degree of incompleteness still persists. So it has been also necessary to deeply check data quality, format and consistency.

In order to fulfill the objectives of the project, we defined a conceptual framework for the linkage of the different datasets, their management, the selection of patients, the definition of the study observation unit, and the comorbidity detection. We did so by creating a multidisciplinary research team, including clinicians with expertise in heart failure and, specifically, covering the entire spectrum of heart failure (from general to advanced/refractory) and of heart failure management modalities (inpatient care: general hospital, specialised referral centre, rehabilitation unit; outpatient care: heart failure clinic, telecardiology services, diurnal hospital for advanced therapies); epidemiologists; ICT experts; statisticians with specific expertise in health data analysis and modelling; management engineers with expertise in studying healthcare services, including economic evaluation. Our scope was to create a flexible platform that should contribute to define subcategories of the broad population of HF patients, not only on the basis of age, gender, and comorbidities, but also on the cluster of primary and secondary diagnosis, in order to help in defining more than one patient profile. Through this framework the criteria of our work are made clear; they guarantee the solidity of the study and could be of use to other project dealing with high-dimensional datasets. In this paper, we will describe the methodological set up of the study, and, in particular, 1) how it was possible to create a reliable database on heart failure using regional administrative data on hospitalizations, emergency services, outpatient care, and drug prescriptions; and 2) how many problems related with dealing with non-integrated data sources were approached, with the aim to build clinically meaningful knowledge from data collected for administrative purposes.

## Methods

### Extraction criteria and evaluation of data quality

With the aim of identifying hospitalizations for HF, we asked Lombardia Informatica s.p.a. (the agency managing the regional data warehouses) to extract data on hospitalizations in Major Diagnostic Categories (MDC) 1, 4, 5 and 11 in the years from 2000 to 2012, and to identify the affected patients, either with single or multiple hospital admissions. Data on hospital admissions of Lombardy residents in other regions for the same MDC were also requested. In-hospital deaths were collected from hospital discharge forms database, while data on out of hospital deaths were retrieved from vital statistics regional dataset.

**Fig. 1:**
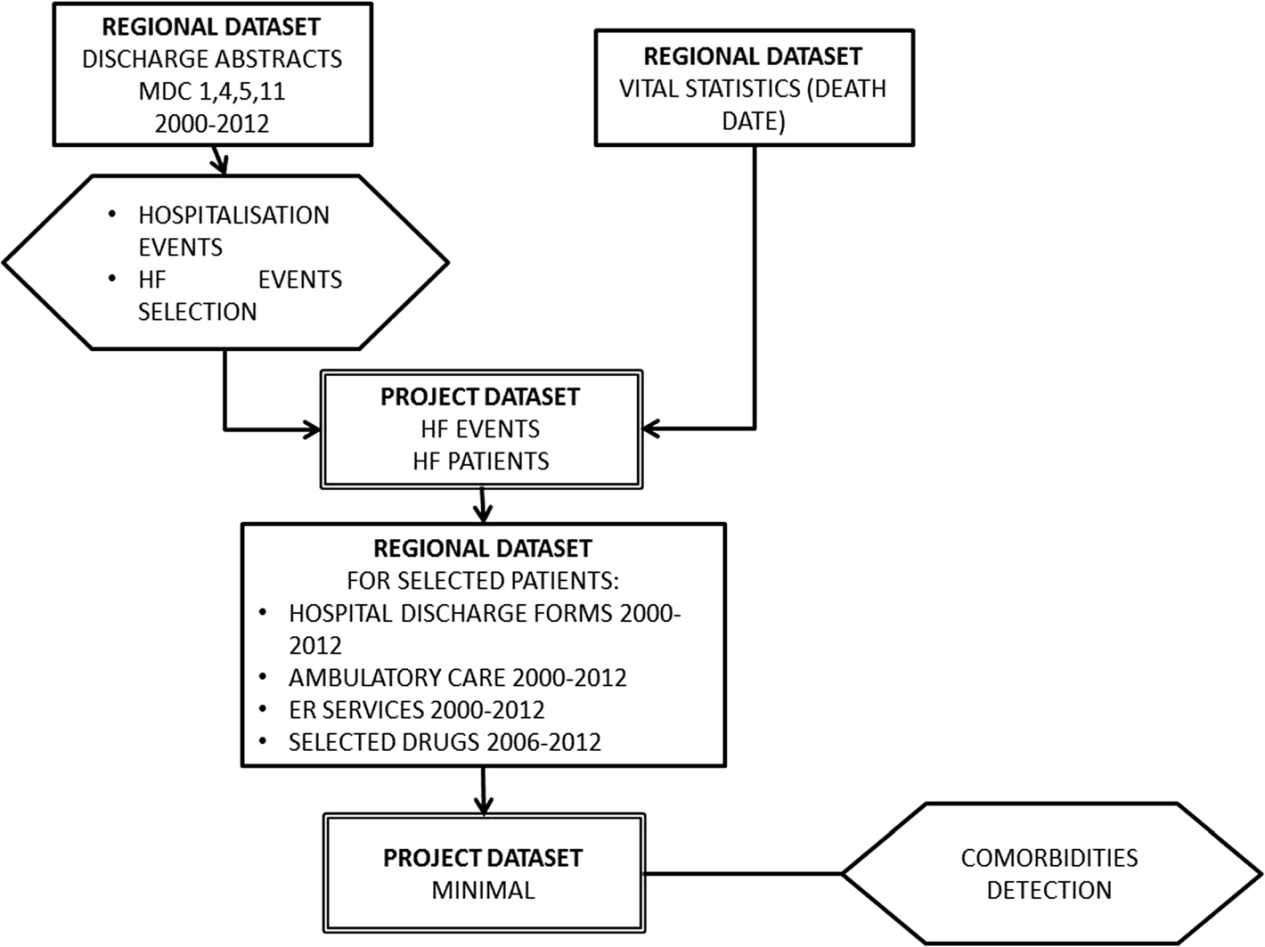
Data processing to construct the project dataset for analysis purposes

Figure 1 shows a scheme of the data sources and data processing utilized to build up the study project database.

A preliminary quality control of the information gathered from the hospital discharge forms had been already performed by the regional administration for reimbursement purposes. However, data quality was reassessed for completeness and consistency using the protocol proposed by the Manitoba Center for Health Policy [22]. The data were then evaluated for accuracy (completeness and correctness) and internal validity (data consistency). The values judged to be invalid were considered as missing.

The presence of an ID (identification) code was used to identify the patient over the years and across the different data sources. The ID code was made anonymous to respect privacy. Records with missing ID were excluded from subsequent analyses, being impossible to keep track of the patient’s history.

### Selection criteria for heart failure hospitalizations

After a comprehensive literature review and an open discussion between epidemiologists, statisticians and clinicians, two criteria were chosen to obtain a complete and accurate selection of HF cases. The criteria were based on:

- indicators proposed by the Agency for Healthcare Research and Quality (AHRQ) [23];
- HF codes as identified by the Center for Medicare and Medicaid Services (CMS) [24], that applies a model of risk adjustment for capitation payment by grouping the diagnosis codes into hierarchical classes (Hierarchical Condition Categories—HCC). The category HCC80 (CMS-HCC80, version 12) refers to heart failure.

Table 1 shows the list of diagnosis codes selected by AHRQ and CMS-HCC criteria. These two different code sets share some common codes for identifying HF, as ‘heart failure’ (428.x), ‘hypertensive heart disease and heart failure’ (402.11), and ‘hypertensive heart disease with kidney disease at stages I–IV and heart failure’ (404.91). Codes selected only by AHRQ are specific of ‘rheumatic heart failure’ (398.90) and ‘hypertensive heart disease with kidney disease at stage V and heart failure’ (403.01), while codes selected only by CMS-HCC model are related to cardio-pulmonary diseases (416.x), cardiomyopathies (425.x) and myocarditis (429.0). Hospital discharge forms with any of the codes included in these lists, in any position, identified HF hospitalizations.

**Table 1:** ICD-9-CM Diagnosis Codes for heart failure used by AHRQ—Quality Indicators and by CMS-HCC (common codes are in bold)

AHRQ codes	398.91-**402.01-402.11-402.91-404.01**-404.03**-404.11**-404.13**-404.91**-404.93**-428.0-428.1-428.20-428.21-428.22-428.23-428.30-428.31-428.32-428.33-428.40-428.41-428.42-428.43-428.9**
CMS-HCC codes	**402.01-402.11-402.91-404.01-404.11-404.91**-415.0-416.0-416.1-416.8-416.9-417.0-417.1-417.8-417.9-425.0-425.1-425.2-425.3-425.4-425.5-425.7-425.8-425.9-**428.0-428.1-428.20-428.21-428.22-428.23-428.30-428.31-428.32-428.33-428.40-428.41-428.42-428.43-428.9**-429.0-429.1

To verify the goodness of these selection criteria, over 750 discharge letters from the three hospitals involved in the project were randomly chosen and analysed, partly by clinicians and partly using text mining techniques [25].

### Definition of heart failure events and of incident cases

The regional database of hospitalizations contains the discharge form from all the admissions within Lombardy. When a patient is transferred from one hospital to another (e.g. from an acute care hospital to a rehabilitation facility, or from a local general hospital to a tertiary care, highly specialized referral center), the regional database contains two administrative records (one from each hospital), but from a clinical perspective they refer to the same episode (HF hospitalization event) within patient's history. Thus, for the purpose of this study, these pairs of discharge units were associated to create single heart failure episode, or event.

New, or “incident” heart failure cases, were identified as patients at their first HF hospitalization. To exclude the patients with prior HF diagnosis, a 5-years period of freedom from HF hospitalization was considered adequate. Therefore, incident cases were identified from 2005 onwards. The choice of a 5 years free from hospitalizations was made because it seems a sufficient enough period to be sure that those was the real first hospitalization for heart failure for that patient and this method was previously used in a paper on myocardial infarction [26].

### Events and patient grouping

Heart failure is a highly heterogeneous condition, affecting mostly -but not exclusively- the elderly, especially women. Thus, various criteria were utilized to characterize HF subgroups, both regarding events and patients.

**Fig. 2:**
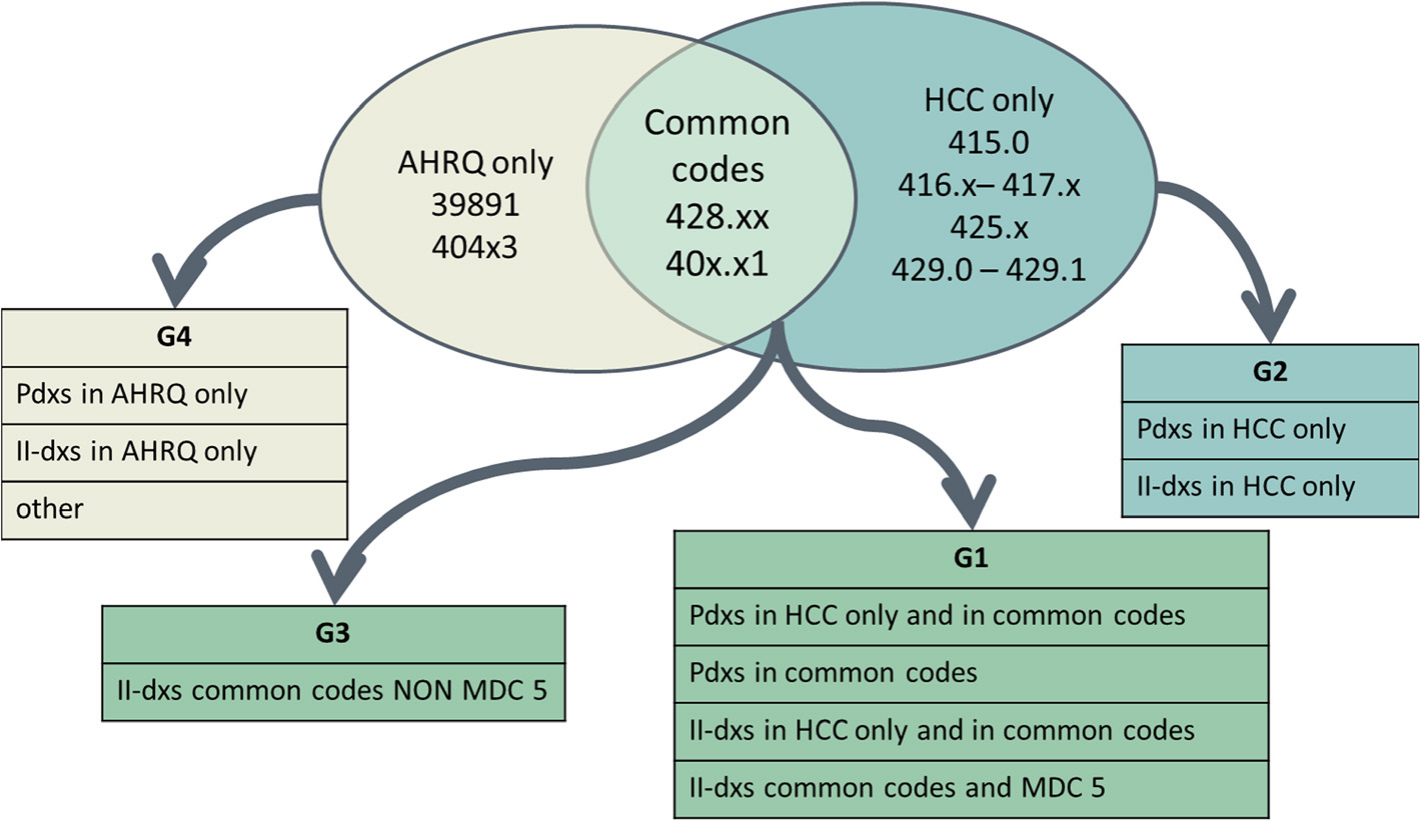
Allocation of patients to groups

- Age: four groups were defined, <18, 18 to 75, 75 to 85, and over 85 years.
- Gender: except for pediatric population (age <18 years), females and males were analyzed separately across all age groups.
- Diagnosis: according to the presence of the AHQR and/or CMS-HCC codes as primary or secondary diagnoses, four different groups were defined, again by agreement between researchers (see Fig. 2 and Table 2). This classification was applied both to each event and to individual patients. For patient characterization, each subject was classified according to the diagnosis Group defined at his/her first heart failure event, independently of subsequent events, if any. In the first three columns of Table 2 we show the criteria for defining the different groups.

**Table 2:** Grouping of adult patients on the basis of selection diagnosis and number of patients belonging to each group

Group	Criteria	Description	nr patients (% of the total)	Sex: nr males (% in the group)	Age: mean (sd) [years]
G1	Main diagnosis in common codes; or secondary diagnosis in common codes and in exclusive HCC; or secondary diagnosis in common codes and at least one admissions of the event in MDC 5	Heart failure was the cause of admission or it complicated another cardiac disease	149,733 (69.07)	70,597 (47.15)	77.76 (11.05)
G2	Main diagnosis in exclusive HCC codes; or secondary diagnosis in exclusive HCC codes	The patient has myocardial or cardiopulmonary disease; no code of cardiac failure reported.	29,515 (13.62)	18,576 (62.94)	69.23 (13.51)
G3	Secondary diagnosis in common codes and no admission to hospital of the event in MDC 5	Acute heart failure was a complication of other disease or chronic heart failure was reported as co-morbidity.	36,802 (16.97)	17,257 (46.89)	80.13 (9.9)
G4	All the other cases	–	732 (0.34)	349 (47.68)	77.46 (12.16)

Common codes = common codes to AHRQ and HCC criteria; Exclusive HCC codes = exclusive codes for HCC criterion

### Identifying and defining comorbidities

Comorbidities were evaluated with the method proposed by Gagne et al. [27], which is a combination of the methods proposed by Romano [28] and Elixhauser [29]. One important detail concerning the recognition of comorbidities is the so-called “look-back period”, i.e., the time prior to the hospitalization that represents the index event. This period must be analysed to intercept comorbidities that may not be reported within the diagnosis list of the current hospitalization event. Sharabiani et al. [30] suggest that a period of 1 year should be sufficient for identifying comorbidities that influence the patient' probability of survival.

A period of 1 year prior to the incident hospitalization was considered for recovering information about patient’s comorbidities at that time. Comorbidities were then updated at each subsequent hospital admission, proceeding in a cumulative way: once a comorbidity had been identified, it was kept throughout the subsequent patient history, regardless of whether it was reported in the diagnosis list of each hospital admission.

### Structure of the database

The project database was built for residents in Lombardy which were hospitalized for heart failure.

It consists in:

1. all hospital admissions for/with heart failure, including rehabilitation and Day Hospital, in-hospital and post-discharge deaths, either occurring at home or in another, subsequent hospitalization, from 2000 to 2012.
2. Outpatient drug prescriptions, provided via the national health service, regarding the Anatomical Therapeutic Chemical (ATC) classes: “Cxxxxxx” (cardiovascular system), “B01xxxx” (antithrombotic agents),”B03xxxx” (anti-anaemia drugs), “A10xxxx” (diabetes drugs), “A01AD05” (acetylsalicylic acid) and “N02BA01” (acetylsalicylic acid-analgesics).
3. data on outpatient services (e.g. blood tests, echocardiography…) and accesses to the emergency department not followed by hospital admission.

A large quantity of data to be processed was thus generated. Therefore, it was necessary to arrange data to make them easily usable, in terms of both management and processing.

This database, that can be called *PROJECT MINIMAL DATASET,* is the result of data gathering and processing summarized in Fig. 1, and was mainly designed to answer the questions: who, what, where, when, how many, and how much.

Table 3 summarises the information fed into the *MINIMAL* database from various data sources. Thus, only some of the fields used in the *MINIMAL* database will be described here, referring to Table 3 t for further information. Each patient is identified by his/her own unique anonymous alphanumerical code across all the data sources. Information about the delivered services is split into three levels. At the greatest detail (third level) this information represents the DRG value for hospital admissions, the regional code for outpatient and emergency room services, and the drug code for pharmaceutical prescriptions. The information concerning “how many” services have been provided varies from source to source. With regard to ordinary hospital admission, it represents the length of stay in hospital, while for the Day Hospital it represents the number of accesses. With regard to outpatient and emergency room services, it is the number of services. For drug prescriptions, it is the number of days of treatment covered by the prescription, based on the number of boxes and the Defined Daily Dose (DDD) for that specific medicinal product. Lastly, all costs refer to the reimbursement provided to the health facility by the national health service.

**Table 3:** Description of construction criteria for *MINIMAL* database

	Ordinary admissions for acute cases	Day Hospital	Outpatient services	Emergency room services	Pharmaceutical prescriptions
Who	Patient ID				
What					
*level1*	Admission for acute cases	Day Hospital	Outpatient service	Emergency room service	Pharmaceutical prescription
*level2*	CCS-principal diagnosis	CCS-principal diagnosis	Service code class	Service code class	ATC code
*level3*	DRG	DRG	Service code	Service code	Drug code
when	Date of discharge	Date of discharge	Date of service delivery	Date of service delivery	Date of prescription
where	Hospital code	Hospital code	ASL code of the hospital	ASL code of the hospital	ASL code of the pharmacy
how many	Length of stay	Number of accesses	Number of services	Number of services	Days covered by prescription according to DDD
how much	Cost of admission	Cost of admission	Cost of the service	Cost of the service	Cost of the prescription

ASL is a territorial grouping of facilities

*Abbreviations*: *ATC* Anatomical Therapeutic Chemical, *CCS* Clinical Classifications Software by Centers for Medicare and Medicaid Services, *DDD* defined daily dose

### Data analysis

The data management and analysis were carried out using the SAS 9.4 software.

## Results

The total number of residents’ admissions occurring from 2000 to 2012 within and outside Lombardy region and classified in MDC 1, 4, 5 and 11 was 6,636,611. 889,060 (13.40 %) records were discarded due to missing patient ID. It is worth noting that the percentage of records without patient ID decreased from 18.87 % in 2000 to 9.04 % in 2012, reflecting a constant improvement of data quality over the years. We observed 812,444 cases of hospitalizations that have been aggregated in 701,701 HF events.

Heart failure admissions were then selected on the basis of AHRQ and/or CMS-HCC heart failure-related diagnostic codes (Table 1), and converted into events when needed, resulting in 701,701 heart failure events from 2000 to 2012, involving 371,766 patients.

The average number of heart failure events per patients was 1.9. During the study period, 229,341 patients (61.69 %) experienced a single event, 71,011 patients (19.10 %) had two events, 31,071 patients (8.36 %) had three events, and 40,343 (10.85 %) had more than three events.

The incident heart failure cases were 217,588 from 2005 to 2012, of whom 216,782 (99.6 %) were adults (age ≥ 18 years) at the time of the first event. Mean age of the entire cohort of incident cases was 73.73 ± 12.5 years, and 107,271 (49.30 %) of them where males. The main demographic statistics for the three groups (G1, G2 and G3) are reported in Table 4. In each group, females were significantly younger than males. Statistically significant differences were found when comparing gender and age composition among groups.

**Table 4:** Demographic statistics for the three groups of patients

	Group 1	Group 2	Group 3
Prevalent cases, 2000–2012			
*N*	489,139	118,087	90,235
% males	66.88	21.16	11.97
Age, males (mean, std dev)	74.20 (10.87)	68.60 (12.30)	77.38 (9.65)
Age, females (mean, std dev)	80.16 (9.70)	73.07 (12.80)	81.71 (9.32)
Incident cases, 2005–2012			
*N*	149,733	29,515	36,802
% males	47.15	62.94	46.89
Age, males (mean, std dev)	74.65 (11.39)	67.20 (13.31)	77.98 (9.940)
Age, females (mean, std dev)	80.55 (9.950)	72.68 (13.15)	82.05 (9.471)

Comorbidities were evaluated in incident cases: 33.62 % patients were from zero comorbidities, 37.52 % had only one comorbidity, 19.89 % had two comorbidities, 6.81% had three comorbidities, and 2.16 % had more than three comorbidities. The most common complications or associated diseases identified at the time of the first event were: hypertension (22 %), lung diseases (18 %), kidney diseases (11 %) and tumors/metastases (6 % and 2 % respectively).

Concerning the text mining of a random sample of discharge letters, the analysis has been performed over 750 letters related to hospitalizations between 2008 and 2012. The text mining system has been trained and evaluated to analyse texts and recognize terms and clinical expressions related to 80 different categories relevant for three objectives of the project: (a) heart failure diagnosis (such as: asthenia, arrhythmias, mitral insufficiency, …), (b) care pathway (such as: aortic counterpulsation, not invasive ventilation, …) and (c) continuity of care (such as: cardiac rehabilitation, disease management, follow-up …). The system performance in automatically annotating the defined categories was evaluated, obtaining an average F-Measure of 0.77 across all the categories of the three objectives. In particular, the average F-Measure in each objective was: (a) heart failure diagnosis: 0.79; (b) care pathway: 0.90; (c) continuity of care: 0.61

As a whole, hospital admissions for any cause- ordinary and Day Hospital, for both acute patients and rehabilitation units, were about 2,7 millions (6.6 GB, text file) in the period from 2000 to 2012. In the same period, the outpatient services totaled almost 129 million (122 GB, text file). Lastly, drug prescriptions amounted to almost 36 million (26 GB, text file). Once all the services delivered to heart failure patients from various sources had been joined together and the ones that occurred after the first heart failure event had been selected, the *MINIMAL DATASET* totaled about 91 million records.

## Discussion

The use of administrative data to study heart failure allows researchers to address the disease in the entire population and for long periods of time. However, studies based on administrative data must be carefully designed, thus may take advantage from an multidisciplinary approach, as indicated, for example in two methodological reviews [31, 32]. This study involved different types of expertise: clinicians, epidemiologists, statisticians, coding experts, and experts of regional datasets and healthcare organization. We think that this mixed research group allowed us to build up a dataset that could be used for different purposes: epidemiology and statistics, clinical characteristics and outcome description, resource utilization, quality and performance indexes, data coding and data quality evaluation, trend analysis, and so on, starting from data which have been collected with different (administrative) purposes.

### Case selection and characterization

Obviously, the criteria for selecting the cases of interest have an important impact on the quality of the study and on the relevance of its results. Several studies [33–35] have addressed the issue of defining the selection criteria for hospital admissions due to heart failure, based on the hospital discharge forms. Saczynski et al. [34] showed that the use of the codes 428.x only is associated with high specificity and positive predictive values, but with low sensitivity. Other authors [36] suggest to expand the set of codes for selecting heart failure-related hospital admissions to increase sensitivity. in order to intercept as much HF hospitalizations as possible, as was done in this study. On the other side, there may be the risk of overestimation and lack of accuracy. For these reasons, two measures we adopted in this study. First, four subgroups of diagnosis codes type and aggregation were defined, of which three (Group 1, 2 and 3) covered almost all the cases, and were then analyzed separately. Group 1 included hospitalizations due to HF, or with HF complicating another cardiac disease or condition, which was the primary diagnosis. Group 2 was characterized by myocardial or cardiopulmonary disease, but decompensated HF was not specifically mentioned either as primary or accompanying diagnosis. Group 3 selected the cases with a non-cardiac primary diagnosis, in whom acute HF represented a complication of another disease or condition, or chronic HF was reported as a comorbidity. Group distribution and basic demographics have been presented here, while outcomes and other details will be described in further papers. At present, we may say that patients had been grouped according to the presence of typical heart failure codes (if absent, a diagnosis code characterising myocardial dysfunction [cardiomyopathy, myocarditis] or cardiopulmonary disease was required), and according to their primary diagnosis (cardiac vs. non-cardiac). Thus, we identified three groups with distinct demographic profiles. This clinically meaningful distinction is of interest also for administrators and auditors: in fact, new discharge forms will be implemented in Italy during the present year, that would distinguish pre-existing secondary diagnosis (proxy of comorbidities) and new-onset conditions (as possible proxy of complications or incident events).

### From events to patients and patient trajectories

A two stage approach was adopted (Fig. 1) in order to first identify heart failure events using hospitalization database, and then to link these data with those concerning drug prescriptions, and outpatients and emergency departments services utilization.

Thanks to the choices made to build the project database, the number of events of care was estimated and referred to events/episodes and to individual patients, avoiding overcounting, and allowing to define correctly the number and time interval of readmissions.

The choice of managing the cases showing more than one sequential admission as a single event was based on the fact that a patient can be treated in different contexts and hospitals for the same HF episode.

A selective criterion was used to define incident cases, that sacrifices the length of the observation period in favor of the accuracy of the identification of newly diagnosed heart failure. For these patients, the linkage of in-hospital and outpatient databases at individual level allows to track patient trajectories according to factors characterizing the incident events and/or to post-discharge management and occurrences.

The *MINIMAL DATASET* obtained as previously described allows interesting analysis at the epidemiological and the economic level.

A key point in patient characterization is the identification of comorbidities and the evaluation of their impact on prognosis. Several papers [28, 29, 37, 38] addressed the issue of determining comorbidities and evaluate comorbidity scores and their prognostic relevance in a context of lack of detailed clinical information. By means of a systematic review, Sharabiani et al. [30] showed that the comorbidity scores proposed by Romano and Elixahauser are among the best ones in predicting long-term mortality [28, 29]. In this study, the method proposed by Gagne et al. [27], that is a combination of the methods proposed by Romano and Elixhauser and shows also a good predictive power of mortality in elderly patients, was used. Moreover, to avoid underestimation of comorbidities, information from hospitalizations within a period of 1 year prior to the incident HF event were recovered, according to recommendations in the literature [30]. For the same reason, we considered a co-morbidity as permanently affecting a patient from the moment it had been diagnosed and thereafter. Although some possibly temporary conditions such as arrhythmias may be overestimated by this “carry on” method, we think that it in more important to avoid the risk of missing relevant chronic comorbidities, such as renal insufficiency or diabetes, that can happen as a result of inaccuracy or different priorities in defining the entire set of primary and secondary diagnosis codes at the end of each hospitalization.

## Conclusions

The methodological principles, criteria, and tips adopted in this study could be used as reference for similar studies for at least 3 reasons:

1. The two steps approach of data retrieval is efficient from a computational point of view and it is useful when researchers are not the primary owners of data;
2. The criteria used in our approach (i.e. case selection, incident event and co-morbidities detection) might be used for other administrative databases;
3. Methods for building the *MINIMAL DATASET* allow the access to a large amount of data, facilitating the approach to analysis.

This work describes the methodological basis of our current research. Some epidemiological results and results on occurrence of heart failure episodes and their cost have been already achieved, and will be presented in subsequent papers. Thanks to the large amount of available data and their completeness, further research will be possible on disease evolution and treatment, and costs of treatment of these patients.

Another interesting area of development refers to the communication of the results of our research; in particular making these results readable by policy makers in order to derive advices on topics useful to steer public health interventions.

## Abbreviations

AHQR Agency for Healthcare Research and Quality; ATC Anatomical Therapeutic Chemical; CMS Center for Medicare and Medicaid Services; DDD Defined Daily Dose; DRG-IDC9-CM Diagnosis Related Groups—International Classification of Diseases, 9th Revision, Clinical Modification; ER Emergency Room; HCC Hierarchical Condition Categories; HF Heart Failure; ID Identification; MDC Major Diagnostic Categories; RCT Randomized Clinical Trials
